# Transitional Remodeling of the Hepatic Extracellular Matrix in Alcohol-Induced Liver Injury

**DOI:** 10.1155/2016/3162670

**Published:** 2016-10-24

**Authors:** Lauren G. Poole, Gavin E. Arteel

**Affiliations:** Department of Pharmacology and Toxicology, University of Louisville Health Sciences Center, Louisville, KY 40292, USA

## Abstract

Alcohol consumption is a common custom worldwide, and the toxic effects of alcohol on several target organs are well understood. The liver is the primary site of alcohol metabolism and is therefore the major target of alcohol toxicity. Alcoholic liver disease is a spectrum of disease states, ranging from simple steatosis (fat accumulation), to inflammation, and eventually to fibrosis and cirrhosis if untreated. The fibrotic stage of ALD is primarily characterized by robust accumulation of extracellular matrix (ECM) proteins (collagens) which ultimately impairs the function of the organ. The role of the ECM in early stages of ALD is poorly understood, but recent research has demonstrated that a number of changes in the hepatic ECM in prefibrotic ALD not only are present, but may also contribute to disease progression. The purpose of this review is to summarize the established and proposed changes to the hepatic extracellular matrix (ECM) that may contribute to earlier stages of ALD development and to discuss potential mechanisms by which these changes may mediate the progression of the disease.

## 1. Introduction

*(1) Alcohol Consumption and Its Impact*. The discovery of fermented beverages was likely accidental and derived from improper food storage. Agrarian culture developed throughout the world, so did the intentional cultivation of crops for alcoholic beverage production. Alcoholic beverages were valued in ancient cultures for several reasons beyond it being a “social lubricant” [1]. In a time when potable water was difficult to acquire, alcohol acted as a relatively safe source of hydration. Additionally, alcohol's modest nutritional value supplemented malnourishment. Furthermore, alcohol had significant medicinal value as an antimicrobial agent. Taken together, the pervasive nature of alcohol consumption throughout the world is unsurprising. Even in cultures that forbid alcohol consumption, the development of such taboos speaks to the fact that these peoples have been exposed to alcohol consumption.

There are many potential benefits of alcohol consumption, as discussed above. Despite these benefits, the idea of a need for moderation in alcohol consumption is as ubiquitous as the consumption of alcohol itself; almost every culture frowns upon public intoxication and alcohol abuse and/or dependence. For example, Aristotle strongly extols the virtue of temperance in his work, “The Nicomachean Ethics.” In modern society, alcohol abuse has an even more significant impact. For example, there is on average more than one alcohol-related driving fatality every hour in the US [2].

In addition to these social consequences, alcohol abuse significantly impacts health. Alcohol requires relatively high concentrations to exert many of its toxic effects in organisms and is therefore, arguably, not an incredibly potent toxin. However, alcohol must be consumed in relatively high doses to cause any noticeable inebriating effects; the legal driving BAC in most US States (0.08% w/v) translates to ~20 mM ethanol. Therefore, the sheer volume of ethanol which humans consume is enough to offset its low potency. In fact, one could argue that alcohol is the most common poison voluntarily consumed at toxic doses by the human population. Chronic alcohol consumption/abuse has been demonstrated to directly damage several organs, including liver [3], lung [4], skeletal muscle and heart [5], the brain [6], and the pancreas [7]. Additionally, alcohol consumption increases the risk of developing several cancers; it is considered a group 1 carcinogen for cancers of the GI tract, liver, breast, and pancreas by the International Agency for Research on Cancer [8]. Ultimately, alcohol consumption is responsible for ~6% of all disability-adjusted life years (DALY) lost in the United States [9], most of which are attributable to alcohol-induced toxicity as opposed to alcohol-related accidents.

*(2) Alcoholic Liver Disease*. The liver is strategically located between the intestinal tract and the rest of the body, making it a critical organ in the clearance of toxins and xenobiotics, including alcohol, that enter the portal blood. The concentrations of alcohol found in the portal blood are much higher than those in the systemic circulation. Additionally, the liver is the primary site of alcohol metabolism, which produces many toxic metabolites. Therefore, it is unsurprising that the liver is a primary target of alcohol toxicity. Although excessive alcohol consumption was associated with organ toxicity since ancient times, the first suggestion that alcohol consumption may directly cause organ damage is credited to Addison in 1836 [10].

Alcoholic liver disease (ALD) affects millions of patients worldwide each year. The progression of ALD is well characterized and is actually a spectrum of liver diseases, ranging initially from simple steatosis, or fat accumulation, to inflammation and necrosis (often called steatohepatitis), and ultimately, to fibrosis and cirrhosis. Although the risk of developing ALD increases in a dose- and time-dependent manner with alcohol consumption [11, 12], only a small fraction of even the heaviest drinkers develop the severe form of the disease, suggesting that other environmental (e.g., HBV or HCV infection) or genetic (e.g., gender or polymorphisms in key genes) factors contribute to overall risk [13]. Clinical management of ALD primarily focuses on maintaining abstinence in the alcoholic and on treating sequelae associated with acute alcoholic hepatitis or cirrhosis [14]. The effects of decompensation (e.g., hepatorenal syndrome) usually lead to the death of the patient, except in the case of a successful liver transplant [15]. Furthermore, the overall risk of developing hepatocellular carcinoma (HCC) increases roughly 20-fold by preexisting cirrhosis, even in patients in which compensation is maintained (i.e., “stable cirrhotics”) [16]; HCC has an even more dismal prognosis than cirrhosis with very high mortality rates [17].

Given the poor prognosis of treating ALD, much of the current research focuses on preventing the development of the disease. Indeed, although the progression of ALD is well characterized, there is no universally accepted therapy available to halt or reverse this process in humans. With better understanding of the mechanism(s) and risk factors that mediate the initiation and progression of ALD, rational targeted therapy can be developed to treat or prevent ALD in the clinics. A potential area of targeted therapy for ALD may be the hepatic extracellular matrix (ECM). Towards this end, the purpose of this review is to summarize the established and proposed changes to the hepatic extracellular matrix (ECM) that may contribute to earlier stages of ALD development and to discuss potential mechanisms by which these changes may mediate the progression of the disease.

## 2. The ECM

**Figure 1:**
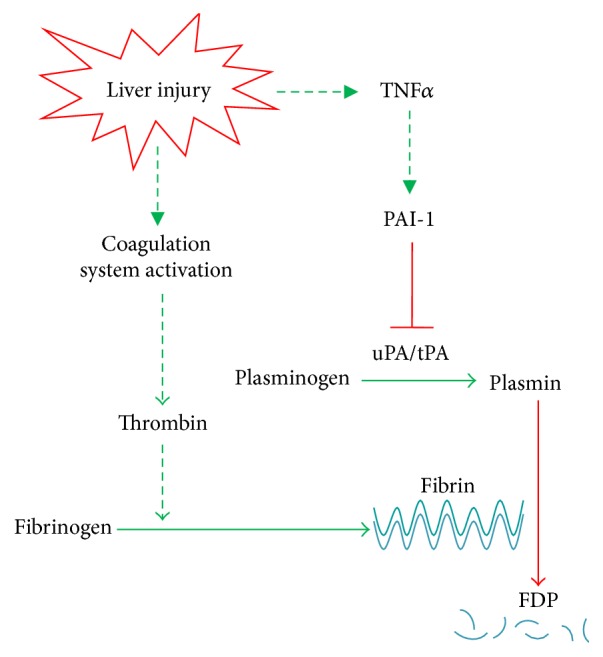
Consequences of transitional ECM remodeling: role of ECM in injury and inflammation. Acute and chronic alcohol exposure causes subtle, prefibrotic alterations to the amount and composition of the hepatic ECM. These changes can impact the overall structure of the ECM, interactions of the ECM with soluble mediators such as growth factors and cytokines, and communication between the cell and the ECM via integrin signaling. Ultimately, these changes contribute to tissue injury and inflammation by several mechanisms, including causing hemostasis, facilitating the migration of inflammatory cells such as neutrophils and monocytes, and activating proinflammatory intracellular signaling cascades.

The extracellular matrix (ECM) consists of a diverse range of components that work bidirectionally with surrounding cells to create a dynamic and responsive microenvironment that regulates cell signaling, recruitment, and tissue function. The basic definition of the ECM comprises fibrillar proteins (e.g., collagens, glycoproteins, and proteoglycans). More recently, groups have extended the definition to include ECM affiliated proteins (e.g., collagen-related proteins), ECM regulator/modifier proteins (e.g., lysyl oxidases and proteases), and secreted factors that bind to the ECM (e.g., TGF*β* and other cytokines) [18]; this broader definition has been coined: the “matrisome” (see below) [19]. The ECM not only provides structure and support for the cells in a tissue, but also acts as a reservoir for growth factors and cytokines and as a signaling mechanism by which cells can communicate with their environment and vice versa [20] (see Figure 1).

### 2.1. ECM (Dys)homeostasis

#### 2.1.1. *De Novo* Synthesis

Under basal conditions, several hepatic cells contribute to the* de novo* synthesis of the ECM, including hepatocytes, epithelial cells in the bile duct (cholangiocytes), and endothelial cells [21]. Although Kupffer cells, the resident hepatic macrophages, do not normally synthesize fibrillar ECM* per se*, they do synthesize several secreted factors (e.g., cytokines) that associate with the ECM (see Section 2.3). The production of ECM components by these cells changes quantitatively and qualitatively in response to injury or stress. Although it is unclear if hepatic stellate cells (HSCs) generate significant ECM during normal tissue homeostasis, activated HSCs transdifferentiate into a myofibroblast-like phenotype and generate ECM in response. Furthermore, other cellular origins of myofibroblast-like cells have been identified, such as periportal fibroblasts, fibrocytes, and transdifferentiated epithelia [22–25]. The contribution of extrahepatic sources to the hepatic ECM via* de novo* synthesis is unclear, but these compartments clearly contribute to ECM via other mechanisms of homeostasis (see below).

#### 2.1.2. Proteolysis

Another major regulation of ECM content is via proteolysis. This regulation can both be at the level of degradation and deposition. Protein families that degrade ECM include matrix metalloproteinases [MMPs; [26]], a disintegrin and metalloproteinases [ADAMs; [27]], a disintegrin and metalloproteinases with thrombospondin motifs [ADAMTS; [28]], cathepsins [29], and plasminogen activators [uPA and tPA; [30]]. Proteases can also regulate the deposition of hepatic ECM; for example, the activation of the coagulation cascade serine proteases (e.g., thrombin) leads to the cleavage of soluble circulating fibrinogen to insoluble fibrin ECM to form a fibrin clot [30]. Another proteolytic cascade that leads to deposition on the ECM is the complement cascade, which has been demonstrated to contribute to experimental alcoholic liver injury [31]. The activity of these proteases is often balanced by protease inhibitors that directly inhibit their activity. For example, MMP activities are inhibited by tissue inhibitors of metalloproteinases (TIMPs) and contribute to collagen accumulation during hepatic fibrosis [32]. Likewise, plasminogen activator inhibitors (e.g., PAI-1) inhibit the activity of uPA/tPA and thereby contribute to the accumulation of fibrin ECM during hepatic injury [see below [30]].

#### 2.1.3. Posttranslational Modifications

Another level of regulation of ECM proteins is via posttranslational modification. This level of regulation contributes to the formation of polymeric fibrils, helical structures, and cross-linked complexes. For example, prolyl 4-hydroxylase hydroxylates terminal proline residues on individual collagen molecules to allow them to form oligomers (*α* chains) and triple helices of collagen fibrils [33]. Recent studies indicate that lysyl oxidases and transglutaminases also contribute to ECM cross-linking [34, 35]. Although these events are important for stabilizing the proteins and preventing their degradation under normal conditions, their activation may contribute to excessive ECM accumulation in response to injury (e.g., fibrosis) [34]. Furthermore, although fibrosis is potentially reversible if the causative insult is removed [36], highly cross-linked ECM may be resistant to resolution [37]. Cross-linking of the ECM may be altered via nonenzymatic means; for example, the formation of advanced glycation endproducts (AGEs) during diabetes is hypothesized to contribute to ECM cross-linking and increased matrix “aging” [38].

### 2.2. ECM and Structure

#### 2.2.1. Physiology

Perhaps the best-characterized function of the ECM is its role as a scaffold, providing support and structure to the surrounding tissue. There are two major components of structural ECM: the interstitial matrix and the basement membrane [39]. Interstitial matrix proteins, including fibronectins, elastin, and fibrillar collagens, form support networks that provide the overall superstructure that shapes and encapsulates the organ. The basement membrane (or* basal lamina*) is a thin sheet of ECM that underlies epithelial and endothelial cells. Similar to the interstitial matrix, the basement membrane comprises collagens, glycoproteins, and proteoglycans that facilitate structure and growth of the cells. In most tissues, the basement membrane is continuous and dense and is a true barrier between the epithelial/endothelial cells and the adjacent parenchymal cell layer. In contrast, the hepatic basement membrane found in the Space of Disse between endothelial cells and hepatocytes is much less dense and is fenestrated. The end result is that, instead of a barrier, this basement membrane more acts as a structural filter and facilitates bidirectional exchange of proteins and xenobiotics between the sinusoidal blood and hepatocytes.

#### 2.2.2. Pathophysiology

Quantitative and qualitative changes to the ECM structure and superstructure can impact overall health of the organ and organism. For example, “aging” of the ECM with increased cross-linking is hypothesized to contribute to dysfunction of several organ systems, including the vasculature [40], heart [41], the eye [42], and most likely the liver [43]. An increase in ECM stiffness can directly impact cellular behavior, such as apoptosis, migration, and proliferation [44], as well as alter shear stress on vasculature cells [45, 46]. Moreover, increased hepatic stiffness associated with ECM changes during fibrosis is hypothesized to contribute to most of the sequelae of decompensation during end-stage liver disease [47, 48] (see Figure 1). Finally, in some cases, ECM proteins may act as autoantigens, that is, host proteins that are capable of activating an immune response. For example, collagen type V (ColV) is an ECM protein that is normally hidden from the immune system. In disease states, such as idiopathic pulmonary fibrosis (IPF), ColV may be “unmasked,” triggering an inflammatory response [49]. A similar phenomenon occurs in atherosclerosis. Although pro-collagen V (a ColV precursor) levels have recently been reported as a biomarker of advanced hepatic fibrosis [50], the role of ColV unmasking in early ALD is unknown and may be a potential target for further investigation.

### 2.3. ECM-Associated Signaling Molecules

A second function of the extracellular matrix is to serve as a reservoir of signaling molecules, including growth factors during development and angiogenesis, as well as cytokines and chemokines during inflammation and disease (see Figure 1). These interactions may serve to present or restrict access of ligands to receptors, modulate the spatial distribution of growth factors, or create chemotactic gradients, or sequester a signaling molecule for later release [51]. One of the best-characterized ECM-associated growth factors is transforming growth factor-beta (TGF*β*). Upon secretion, TGF*β* binds to latency associated peptides (LAPs), which associate with latent TGF*β* binding proteins (LTBPs). These LTBPs interact with several different ECM and ECM-associated proteins, including vitronectin and fibronectin. The interaction of LTBPs with ECM proteins forms large latent complexes (LLCs), which prevent active TGF*β* from interacting with cell surface receptors. TGF*β* can then be liberated from the matrix by a variety of proteases, including MMP activity, plasmin, urokinase, and thrombin [52].

The extracellular matrix also participates in the inflammatory response by associating with several different cytokines and chemokines. Tumor necrosis factor-alpha (TNF*α*) is a major inflammatory cytokine that is known to associate with the ECM. In the early 1990s, Lider and colleagues demonstrated that TNF*α* associates with both fibronectin and laminin [53, 54]. They found that TNF*α* bound to both of these ECM proteins enhances the binding of CD4+ T-cells to the matrix, thereby facilitating the infiltration of these inflammatory cells into the tissue. More recently, it was demonstrated that fibronectin-associated TNF*α* affects the migration of T-cells toward a chemotactic stimulus by acting as a “stop” signal for the migrating cells [55]. Fibronectin-bound TNF*α* also influences the migration of other inflammatory cell types, such as monocytes and macrophages [56]. This study found that FN-associated TNF*α* stimulates monocytes to secrete MMP9, which is critical for the migration of monocytes through the ECM.

In addition to the potent proinflammatory cytokine TNF*α*, chemokines are also known to associate with the ECM. Interleukin-8 (IL-8) is a potent neutrophil chemotractant that is known to associate with glycosaminoglycans (GAGs) of the ECM, particularly heparin, chondroitin-6-sulfate, chondriotin-4-sulfate, dermatan sulfate, and hyaluronan. The interaction of IL-8 with these GAGs is critical for the chemotractant capability of IL-8. Mutant IL-8 lacking the C-terminal GAG binding domain does not recruit neutrophils to the same extent as WT IL-8 [57]. Taken together, the results of these studies suggest that the interaction of cytokines and chemokines with the ECM is critical for migration of inflammatory cells to the site of tissue damage, permitting both the clearance of infection and the propagation of the inflammatory response.

### 2.4. Cell/Matrix Interactions

As mentioned previously, the ECM is more than simply a passive scaffold acting to support the cells in a tissue (see Figure 1). The ECM is also a dynamic signaling molecule that allows the environment to interact with the cell and the cell to interact with the environment. One family of receptors that mediate these interactions is the integrins. Integrins are heterodimeric proteins composed of *α* and *β* subunits, with at least 24 different combinations having been identified in mammalian cells [58]. Integrins transfer information from the ECM to the cell, allowing rapid and dynamic responses to changes in the extracellular environment. Integrins play a myriad of roles within the body, including proliferation/angiogenesis and maintenance of differentiation, as well as inflammation and apoptosis [59, 60]. Integrins are found on almost all cell types in the liver, and dysregulated integrin signaling has been demonstrated to be involved in hepatic fibrogenesis in a wide variety of liver diseases, as well as inflammatory liver injury [61]. Altering the composition of the ECM has the potential to alter inflammatory signaling in liver via a myriad of mechanisms.

There are several key integrins known to be involved in alcoholic liver disease. First, the *β*_2_ family of integrins is a key regulator of alcohol-induced liver inflammation. Neutrophilic inflammation is a hallmark of alcoholic (steato)hepatitis in both humans and animals [62]. Neutrophil transmigration and extravasation into the hepatic tissue is mediated largely by the activation of *β*_2_ integrins [62]. There are several key *β*_2_ integrins involved in leukocyte migration and trafficking, all sharing the common *β* subunit CD18. There are four *α* subunits that associate with CD18, forming the integrins lymphocyte function-associated antigen-1 (LFA-1, *α*_L_ *β*_2_), macrophage-1 antigen (Mac-1, *α*_M_ *β*_2_), also known as compliment receptor-3 (CR-3), *α*_X_ *β*_2_, and *α*_D_ *β*_2_ [61]. Neutrophils adhere to hepatocytes ICAM-1 via *β*_2_ integrins, particularly Mac-1, and this adhesion is associated with tissue damage by upregulation of oxidative stress pathways [62]. Inhibition of CD18 using an anti-CD18 antibody was demonstrated to be protective against liver inflammation in rats exposed to a chronic alcohol diet [63].

A second family of integrins, the *β*_1_ integrins, has also been associated with alcohol-induced liver injury. The *β*_1_ subunit associates with 10 different *α* subunits which bind a wide variety of ligands, including collagen, laminin, fibronectin, and vitronectin [61]. One study in rats demonstrated that ethanol feeding caused a downregulation of *β*_1_ subunit expression in perivenular hepatocytes and that this downregulation impaired the ability of the hepatocytes to adhere to several different ECM substrata [64]. The results of this study suggest that an ethanol-mediated loss in hepatic integrin expression may be one mechanism by which alcohol impairs communication of the hepatocyte with the extracellular environment and, by extension, normal homeostatic processes. Alternatively, recent research has implicated a proinflammatory role for different *β*_1_ integrins in alcoholic hepatitis. Specifically, hepatic neutrophil recruitment in a rat alcoholic hepatitis model was found to be mediated by interactions between neutrophils *α*_4_ *β*_1_ and *α*_9_ *β*_1_ and the ECM protein osteopontin [65]. The role of osteopontin in ALD will be discussed in greater detail in Section 3.2. Taken together, these examples illustrate that alcohol causes an altered signaling pattern between the cell and its extracellular environment, leading to both the parenchymal cell injury and inflammatory cell infiltration that are hallmarks of alcoholic liver injury.

There are also several nonintegrin receptors involved in signaling between the ECM and the cell. CD44, a type I transmembrane glycoprotein with over 20 different isoforms, has been demonstrated to be involved in liver disease and inflammation. Patients with ALD, including alcoholic steatosis, alcoholic hepatitis, and alcoholic cirrhosis, in fact have elevated CD44 expression [66]. The canonical CD44 ligand is hyaluronic acid (HA). Interactions between this ECM glycosaminoglycan and CD44 are known to facilitate migration of leukocytes to inflamed tissue, as well as the progression of inflammatory injury [67]. Alternatively, CD44 has been implicated in the resolution of injury by facilitating the migration of hematopoietic stem cells to the injured liver [68]. CD44 interactions with HA are therefore an interesting point for further investigation. CD44 also binds the ECM protein osteopontin. The role of osteopontin in ALD will be discussed in greater detail in Section 3.2. Interestingly, osteopontin binding via CD44 has been shown to activate hepatic stellate cells, even at relatively low concentrations of ethanol (10 mM) [66]. In addition to the integrin receptors previously covered, CD44 is a key nonintegrin receptor for further investigation as a target in ALD.

## 3. ECM Remodeling in ALD

### 3.1. Fibrosis: Beyond Collagen

When an organ is chronically damaged from multiple hits, this injury often overwhelms the ability of the organ to recover and rebuild from the damage. Under such conditions, the organ often remodels in response to the damage. Even in the case of liver, which is well known for its ability to regenerate, remodeling (i.e., fibrosis) is a common response to chronic inflammatory liver injury. The fibrotic stage of disease has traditionally been characterized by robust remolding of the hepatic ECM, particularly deposition of collagen type I in the hepatic sinusoids by hepatic stellate cells (HSCs). The accumulation of collagen ECM in the hepatic sinusoids drastically alters the architecture of the organ, leading to the development of cirrhosis and its associated complications (see Section 2.2). However, the matrisome of the healthy and diseased liver is significantly more diverse than collagen ECM. It has become increasingly understood that the traditional model of hepatic fibrosis, with its focus on collagen type I, is incomplete. Specifically, it is more recently understood that many other ECM proteins are altered in hepatic fibrosis, such as laminin, fibronectin, and fibrin(ogen) [69]. The impacts of these other qualitative and quantitative changes to disease development are incompletely understood.

### 3.2. Transitional Changes to the ECM: Before Fibrosis

**Figure 2:**
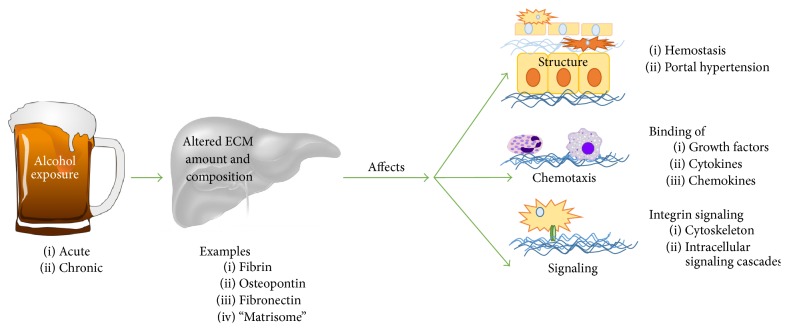
Transitional remodeling of the hepatic ECM in ALD. Acute injury, such as acute (binge) alcohol exposure or low levels of alcohol exposure, causes formation of a transitional extracellular matrix through several known mechanisms, such as activation of the coagulation cascade. This transitional ECM, while it causes no overt changes in the architecture of the organ, may contribute to injury and inflammation. If the insult is removed, the transitional ECM may resolve back to a normal state. With continued disease progression, the transitional matrix may progress to a fibrotic matrix via increased ECM synthesis and blunted ECM degradation, ultimately leading to decreased liver function.

As discussed above (see Section 2), the hepatic ECM is a complex system that responds dynamically to stress. Proteomic-based studies in other organs have demonstrated that the matrisome responses dynamically in composition after insult well before fibrotic changes to the organ [70–72]. These changes to the ECM may not alter overall ECM architecture and are therefore histologically undetectable. Nevertheless, these changes have potential to alter hepatic phenotype and function (see Section 2) [73]. These acute responses can be viewed as an arm of the wound healing response and facilitate recovery from damage, which resolves once the damage is repaired. However, under conditions of chronic injury, these changes contribute to activation of a significant remodeling response that leads to scar formation (i.e., fibrosis) (see Figure 2).

#### 3.2.1. The Coagulation Cascade and Fibrin ECM in ALD

**Figure 3:**
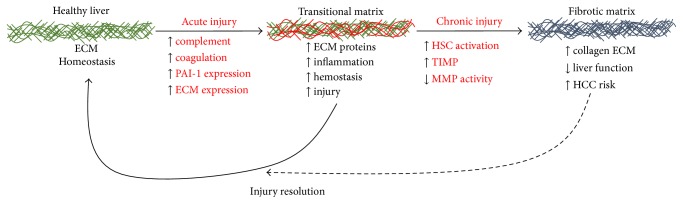
Alcohol-induced liver injury activates the coagulation cascade. Activation of the coagulation cascade contributes to liver injury in models of both acute and chronic alcohol exposure. Alcohol promotes the deposition of fibrin ECM by both increasing its formation (thrombin cleavage of fibrinogen) and preventing its degradation (PAI-1 induction). Fibrin matrices can be proinflammatory by causing hemostasis, acting as an adhesive substrate for migrating inflammatory cells, and altering integrin signaling.

The fibrin coagulation system is largely regulated by the liver via two pathways: coagulation and fibrinolysis (see Figure 3) [74]. Activation of the coagulation cascade induces thrombin-mediated cleavage of fibrinogen to fibrin, leading to the deposition of a fibrin clot. On the other hand, breakdown of the fibrin clot (fibrinolysis) is mediated by plasmin. Plasminogen activator inhibitor-1 (PAI-1) is a protease inhibitor that prevents the conversion of plasmin to its active form. Therefore, inhibition of fibrinolysis by PAI-1 can accumulate fibrin ECM, even in the absence of enhanced fibrin deposition by the thrombin cascade [75]. Dysregulation of the coagulation cascade and fibrinolysis in the setting of hepatic injury results in the formation of fibrin clots in the hepatic sinusoids [76, 77], causing microregional hypoxia and ultimately hepatocellular death [78, 79]. Fibrin ECM is not simply an inert physical structure; it also binds/interacts with several cellular biomolecules. For example, fibrin matrices are known to interact with integrin receptors, including integrin *α*_V_ *β*_3_ via its arginine-glycine-aspartic acid- (RGD-) binding motif [61]. Blocking the interaction between fibrin ECM and integrin *α*_V_ *β*_3_ has been shown to protect against acute alcohol-induced liver injury and inflammation, with no effect on fibrin accumulation itself [80].

Elevated PAI-1 levels and impaired fibrinolysis are common during the development of ALD [81]. Indeed, elevated PAI-1 levels during disease development are associated with later disease severity [82]. In fact, clinical data from human patients support the hypothesis that PAI-1 plays a critical role in ALD [83]. However, few clinical studies have focused on inhibiting PAI-1 as a potential therapy to slow the development of ALD. Indeed, there is increasing understanding that cirrhosis is a hypercoagulable state; both bleeding and thrombosis are commonly associated with end-stage liver disease [84]. In light of this, it is fascinating that anticoagulation therapy using enoxaparin was reported to prevent decompensation in cirrhotics with portal vein thrombosis [85].

#### 3.2.2. Osteopontin

Osteopontin, also designated secreted phosphoprotein-1 (SPP-1), predominantly serves as an extracellular structural glycoprotein. Its synthesis is greatly upregulated in human ALD and in animal models and has been linked to activation of HSC and liver fibrosis [86] and with poor outcomes in alcoholic hepatitis [87]. Additionally, as previously mentioned, osteopontin-mediated neutrophil chemotaxis via integrin interactions has been demonstrated to directly contribute to the development of alcoholic hepatitis in rat models [65]. However, more recent work with genetically modified mice has indicated that overexpression of osteopontin prevents early ALD, most likely via binding LPS [88], and knockout of osteopontin promotes the neutrophilic infiltration of the liver in a model of alcoholic hepatitis [89]. In contrast, osteopontin appears to exacerbate experimental hepatic fibrosis, at least in part by delaying fibrosis resolution [90]. Additionally, osteopontin also interacts with cells via the integrin receptor *α*_V_ *β*_3_. Elevated expression of this integrin receptor is associated with increased osteopontin levels in human alcoholic cirrhosis, as well as in experimental alcohol administration in mice. Moreover, inhibition of this integrin in cultured hepatic stellate cells blunts stellate cell activation after alcohol exposure [66]. The roles of osteopontin at various stages of ALD are thus open to further investigation.

#### 3.2.3. Fibronectin

Fibronectin is a major extracellular matrix glycoprotein found at high levels in many tissues, including the liver. In fact, one form of fibronectin, plasma fibronectin (pFN), is the most abundant ECM protein in the liver. Alternatively, the other form of fibronectin, cellular fibronectin (cFN), is found at low levels in the liver in the pericellular matrix. It is this form of fibronectin, cFN, that has been primarily linked to the progression of ALD [extensively reviewed in [91]]. For example, one study demonstrated that chronic alcohol feeding enhanced hepatic cFN deposition within the first 8 weeks of ethanol feeding and that increased levels of cFN preceded activation of hepatic stellate cells [92]. These results indicate that cFN in the transitional ECM may act as a biomarker of early-stage ALD. Additionally, hepatocytes from ethanol-fed rats show impaired degradation of exogenously administered cFN, providing one potential mechanism by which cFN accumulates in the alcohol-exposed liver. Furthermore, although exposing cells to cFN did not affect cell viability, administration of cFN to hepatocytes stimulated enhanced expression of inflammatory cytokines, such as TNF*α* and IL-6 [93]. Increased concentrations of cFN may also directly stimulate Kupffer cells to produce a robust inflammatory response. For example, one study showed that rat primary KCs enhanced expression of the proinflammatory cytokines TNF*α* and IL-6 in response to cFN exposure [94]. Taken together, these results show that fibronectin is a key component of the transitional ECM which forms in response to ethanol feeding. Enhanced levels of cFN are detected in prefibrotic ALD and may, in fact, contribute to the progression of the disease.

#### 3.2.4. The Matrisome

As detailed in the examples above, previous studies have shown that subtle changes in the ECM may contribute to the development of liver injury, potentially well before significant histologic changes (e.g., fibrosis). However, the research in the liver field up to this point has generally been restricted to study of single ECM proteins (e.g., fibrin) [76, 95]. Given the dynamic nature of the “matrisome,” which comprises over 1000 fibrillar ECM proteins (i.e., “core matrisome”) and other biomolecules associated with the ECM [18], discovery-based approaches may yield new information. A more ‘omic approach has been previously hampered by the difficulties associated with the low abundance and insolubility of many ECM proteins. This limitation has been overcome by sample preparations designed to specifically solubilize the ECM [18]. Such approaches could provide new information on the impact of ALD, at all stages, on the matrisome, and by extension may identify new druggable targets to prevent and/or treat the disease.

## 4. Summary and Conclusions

The hepatic ECM and associated biomolecules comprise a dynamic compartment. Homeostasis of the matrisome is critical, for not only overall structure of the organ, but also function. Dyshomeostasis of the matrisome is affiliated with all stages of chronic liver disease, including alcoholic liver diseases. Whereas the impacts of this compartment on liver disease are well understood in some contexts (e.g., fibrosis and cirrhotic decompensation), there are still critical gaps in our understanding that could/should be filled. Importantly more recent ‘omic approaches to explore changes to the matrisome may lead to new discoveries, biomarkers, and/or druggable targets to treat ALD.
